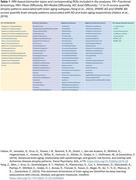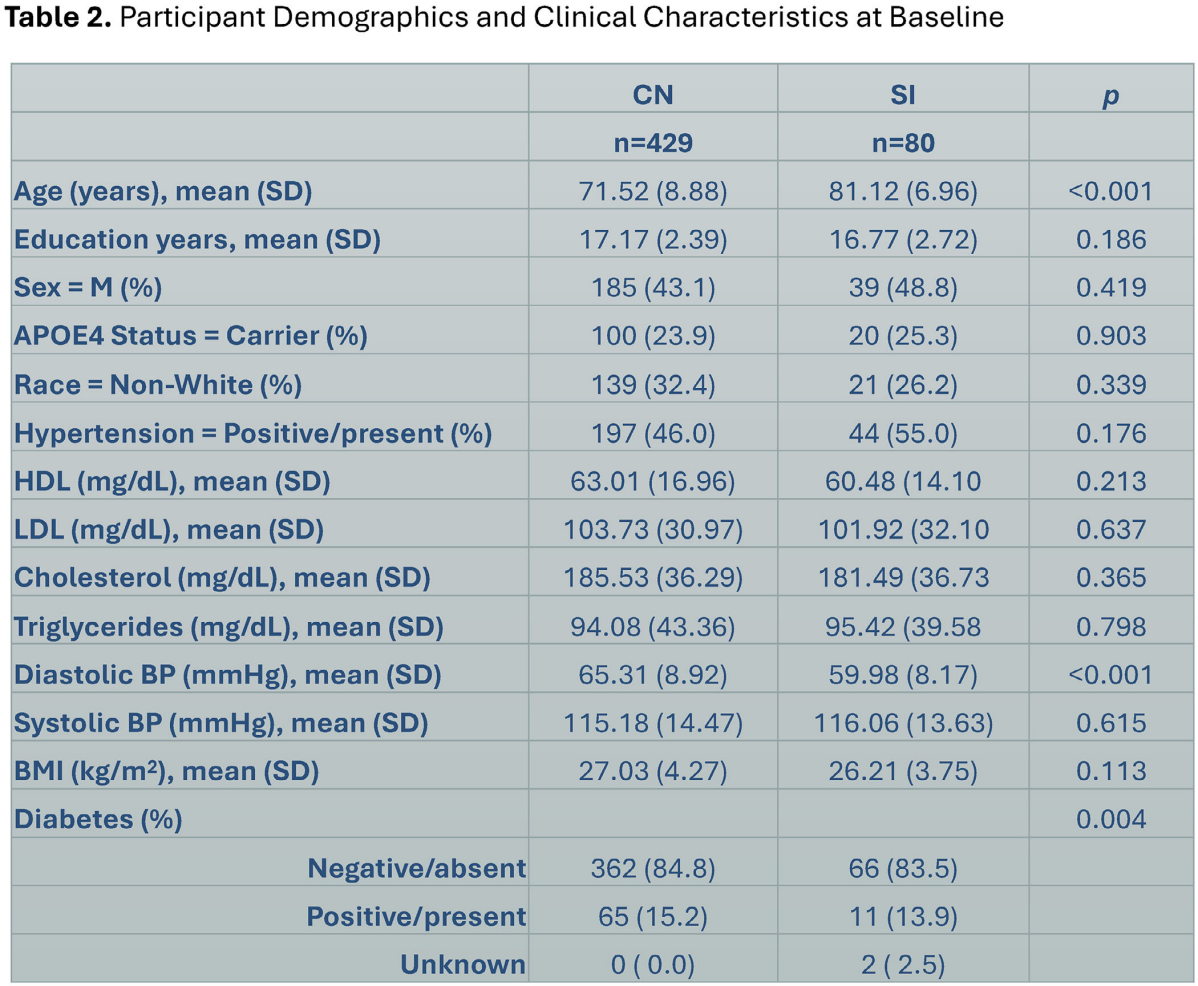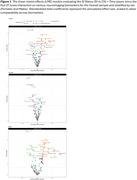# Comparing Macro‐ and Micro‐structural Predictors of Subsequent Cognitive Impairment in the BLSA

**DOI:** 10.1002/alz70856_100392

**Published:** 2025-12-24

**Authors:** Tugce Duran, Murat Bilgel, Yang An, Sridhar Kandala, Christos Davatzikos, Bennett A. Landman, Guray Erus, Keenan A. Walker, Susan M. Resnick

**Affiliations:** ^1^ National Institute on Aging, National Institutes of Health, Baltimore, MD, USA; ^2^ Laboratory of Behavioral Neurosciences, National Institute on Aging Intramural Research Program, National Institutes of Health, Baltimore, MD, USA; ^3^ Center for AI and Data Science for Integrated Diagnostics, University of Pennsylvania, Philadelphia, PA, USA; ^4^ Vanderbilt University Institute of Imaging Science, Vanderbilt University Medical Center, Nashville, TN, USA; ^5^ Artificial Intelligence in Biomedical Imaging Laboratory, Perelman School of Medicine, University of Pennsylvania, Philadelphia, PA, USA

## Abstract

**Background:**

Neuroimaging biomarkers offer valuable insights into the development of MCI or dementia. Recent evidence from the Baltimore Longitudinal Study of Aging (BLSA) Neuroimaging cohort suggests that differences in structural and functional changes over time may provide reliable markers of progression, particularly in subsequently impaired (SI) older adults. However, further research is essential to identify the most sensitive markers of SI during the preclinical stages. This study investigates MRI‐based macro‐ and micro‐structural predictors that distinguish SI from cognitively normal (CN) older adults.

**Method:**

The cohort included 509 CN BLSA participants aged 50+ who had longitudinal cognitive assessments, including adjudication for cognitive status, and 3T MRI scans. MRI‐based metrics included DTI parameters (FA, MD, RD and AD) for white matter (WM) integrity, cortical thickness, regional volumes, and machine learning‐derived atrophy scores. A total of 154 MRI‐based biomarker ROIs were examined (Table 1). Of the 509 CN, 80 individuals developed SI during follow‐up (median time to SI: 4.6 years). Linear mixed‐effects models were used to examine the associations between cognitive status and longitudinal MRI biomarkers, adjusting for baseline age, sex, education years, APOE e4 status (APOE e4 carrier vs. non‐carrier), and race. Models with regional volumes were also adjusted for ICV at age 70.

**Result:**

Table 2 lists the baseline demographic and clinical characteristics for CN and SI individuals. Longitudinal analyses revealed significantly faster declines in DTI WM tract measures and cortical thickness in SI compared to CN individuals, with SI males primarily driving the WM changes in commissural and association tracts and SI females showing greater atrophy in occipital regions (Figure 1).

**Conclusion:**

The observed findings emphasize the utility of selected DTI and cortical thickness measures as sensitive markers of early changes in brain integrity. Further, we found sex‐specific patterns in trajectories associated with cognitive status, with SI females showing more pronounced macrostructural brain changes in cortical regions and SI males exhibiting more significant changes in WM microstructural integrity. Our study highlights the importance of sex‐stratified analyses in identifying early brain changes that predict later cognitive impairment.